# Supplementary material for: Enablers and barriers to effective HIV self-testing in the private sector among sexually active youths in Nigeria: A qualitative study using journey map methodology
Source: PLoS One. 2023 Apr 27;18(4):e0285003. doi: 10.1371/journal.pone.0285003 (PMC10138200; doi:10.1371/journal.pone.0285003)
Supplement: S1 Appendix — (DOCX) [file pone.0285003.s001.docx]

## Appendix I: IDI Guide, Sexually Active Males and Females

**Introduction and Consent**

My name is (xxxxx). I am from Busara Center for Behavioral Economics. Thank you for agreeing to take the time to speak with us today. Is now still a good time to speak?

Thank you. We are conducting research with SFH on HIV self-testing in the private sector. We will be asking questions on a broad range of topics, including your opinions and possible experiences with these kits. This interview will take approximately 30-40 minutes.

I want this to be an open discussion, so please feel comfortable sharing with us what you think. There are no right or wrong answers, and you are welcome to answer in any way you like. If there is anything you are not comfortable answering, please let us know. You are welcome to stop the interview at any time.

We would like to record the session on an audio recorder, so that we can go back and write down what you said later. The notes and recordings will only be kept by the researchers. We will keep any information that could identify you personally separate from those notes.

1. Are you comfortable with this and willing to be interviewed?
2. I would like to record the conversation. Do you consent to this?
3. Do you have any questions for me?
4. Are you ready to start?

**Icebreaker and Demographics**

[Objective: Ensure the respondent is comfortable, chit chat a little bit and share information about yourself too.]

1. Before we start I am curious to know a bit more about you. How was your day so far, and what did you do before we met?
2. How old are you?
3. Do you study or work?
4. [If they work] What do you do for a living?
5. As already mentioned, we are talking about HIV Self-Testing today, that’s HIVST in short.
   1. Do you know what HIV self-testing is?
   2. Have you seen adverts for HIV self-testing?
   3. Have you spoken about HIV self-testing with friends or family?
   4. Have you ever used a HIV self-test kit?

**Section A: Users**

Awareness and Demand

1. How did you first learn about HIV self-testing?
2. Did someone come and speak to you about HIVST, or did you actively seek information yourself?
   1. [If from a third party]
      1. Who told you about HIVST?
      2. Did you trust this information? Please explain.
   2. [If they sought information themselves]
      1. Where did you search for information? Please explain.
      2. What made you search for information? Please explain.
3. Do you know if any of your friends or peers know about the HIVST kits?
   1. [If yes] What do they think about the kits?

Take Up

1. Where did you buy or obtain the HIVST kit? (i.e., did they buy it from a pharmacy, or did they get it from a community health worker, at a health care facility, through online ordering or any other sources or channel? Please explain sources.)
2. Is this the most convenient way for you to get the HIVST kit? Please explain.
3. Why did you eventually decide to buy [or take] the HIVST kit?
4. Was this a hard decision for you to make? Please explain.
5. [For those who purchased the kit at a pharmacy] What did you think about the price for the self-test kit at the pharmacy? (i.e., too high or just right)
   1. How much would you be willing to pay for the HIVST kit?
6. [If they did not get the kit at a pharmacy] Would you feel comfortable getting an HIVST kit at a [Uganda: drug store/Nigeria: PPMVA] (patent and proprietary medicine vendors)? Please explain.

Use

1. Please tell me about the first time you used an HIVST kit.
   1. Did you think the test was easy or hard to use?
   2. Did you use it by yourself, or was someone there to assist you?
   3. Did you receive additional information on how to use the kit from any of the following sources: provider, distributor, pharmacist, leaflet (outside the instructions for use inside the self-test kit), online information, chatbot? Which ones?
   4. What instructions or other materials were provided to help you use the kit properly?
      1. Did you find these materials helpful?
   5. Where did you use the self-test kit?
   6. Did you trust the results?
   7. Did you feel confident interpreting the results with the instructions provided?
2. Where would you go or who would you ask if you had questions on using the HIVST kit?
3. Would you use an HIVST kit again in the future? Please explain.
4. Would you recommend HIV self-testing to a friend? Please explain.

Linkage to Care and Reporting

1. When purchasing [or obtaining] the kit, did you receive any information on next steps for what you should do after using the HIVST kit?
   1. [If yes] Where did you get this information?
   2. What did they tell you?
   3. Was the information clear to you? Please explain.
2. Would you mind telling me what you did after you used the HIVST kit?
   1. Did you report or register your results anywhere?
      1. [If yes] Where did you report your results?
      2. How did you know to report your results using this process?
   2. Did you tell your results to anyone? Whom?
3. [If they have not reported back their results] Would you mind telling me what stopped you from reporting back your results?
4. If you were to report your results, what do you think the potential benefits could be?
   1. What do you think the potential negative consequences could be?
5. If your test result was reactive, did you go for confirmative testing?
   1. Where did you go?
   2. When did you go for confirmative testing?
   3. What was the outcome of the confirmative testing?
6. If the test result was negative, did you seek information on HIV prevention, or did you go to access prevention services (e.g., VMMC or PrEP)?
   1. Where did you go?
   2. When did you go?
   3. Which services did you take up?
7. How were your experiences with these services [either confirmatory testing or HIV prevention services]?
   1. What could have been better and of help for you?

**Section B: Non-Users**

Awareness and Demand

1. Have you ever heard about HIVST kits?
   1. [If yes] Can you still remember what you thought when you first learned about them?
      1. What were your emotions and thoughts?
   2. [If yes] Did someone come and speak to you about HIVST kits, or did you actively seek information yourself?
      1. [If from a third party]
         1. Who told you about HIVST kits?
         2. Did you trust this information? Please explain.
      2. [If they sought information themselves]
         1. Where did you search for information? Please explain.
         2. Why did you decide to search for information on HIVST kits?
2. Do you know if any of your peers, friends, or family know about the HIVST kits?
   1. [If yes] What do they think about the kits?
3. What would motivate you to take up, buy, and use an HIVST kit?
   1. Is there any particular reason why you haven’t used an HIVST kit before?

Take Up

1. Where would you go if you wanted to buy or obtain an HIVST kit? Please explain.
2. Would you feel comfortable buying an HIVST kit at a Pharmacy, PPMV, or from a Lab scientist? Please explain.
3. Do you know the price of HIVST kits?
   1. [If yes] What do you think of the price? (i.e., too high, or just right?)
   2. How much would you be willing to pay for an HIVST kit?

Use

1. Suppose you purchased an HIVST kit. Where would you go or who would you ask if you had questions on using the kit?
2. Do you have any experience using self-test kits, such as malaria self-test kits [or pregnancy tests for women]?
   1. [If yes] What has been your experience using these self-test kits?
      1. How often do you use these kits?
      2. Do you find them easy to use?
      3. Do you trust the results provided by these kits? Please explain.
      4. Could you please describe the steps you follow after using the kits?
3. [If they used self-test kits in the past] How do you think the self-test kits you have used in the past would compare with HIVST kits?
   1. Would you trust the results more or less?
   2. Would you be willing to pay as much for an HIVST kit as you would for these other self-test kits?
   3. Do you think the HIVST kits would be easier or harder to use?
   4. Would you know what to do after taking the test?

Linkage to Care and Reporting

1. Suppose you took an HIV self-test. What would be your next steps after receiving the test results?
   1. Would you seek confirmatory testing from your healthcare provider? Please explain.
   2. Would you seek HIV prevention services, such as PReP or VMMC? Please explain.
