# Supplementary material for: Enablers and barriers to effective HIV self-testing in the private sector among sexually active youths in Nigeria: A qualitative study using journey map methodology
Source: PLoS One. 2023 Apr 27;18(4):e0285003. doi: 10.1371/journal.pone.0285003 (PMC10138200; doi:10.1371/journal.pone.0285003)
Supplement: S2 Appendix — (DOCX) [file pone.0285003.s002.docx]

## Appendix II: FGD Guide, Sexually Active Males and Females

**Introduction and Consent**

My name is (xxxxx). I am from Busara Center for Behavioral Economics. Thank you for agreeing to take the time to speak with us today.

Thank you. We are conducting research with SFH on HIV self-testing in the private sector. We will be asking questions on a broad range of topics, including your opinions and possible experiences with these kits. This interview will take approximately 60 minutes.

I want this to be an open discussion, so please feel comfortable sharing with us what you think. There are no right or wrong answers, and you are welcome to answer in any way you like. If there is anything you are not comfortable answering, please let us know. You are welcome to stop the interview at any time.

We would like to record the session on an audio recorder, so that we can go back and write down what you said later. The notes and recordings will only be kept by the researchers. We will keep any information that could identify you personally separate from those notes.

1. Are you comfortable with this and willing to be interviewed?
2. I would like to record the conversation. Do you consent to this?
3. Do you have any questions for me?
4. Are you ready to start?

**Icebreaker and Demographics**

[Objective: ensure the respondent is comfortable, chit chat a little bit and share information about yourself too.]

Let us start by discussing a few ground rules to ensure that this discussion is as engaging as possible for everyone.

1. Ensure that your mobile phone is turned off or on silent mode during the discussion.
2. Ensure that you do not use your mobile phone to record any videos or photos of this discussion.
3. Please try to protect each other’s confidentiality. Feel free to share what you heard here but do not link it back to someone here specifically. Some of you may know each other. Even if you do, please respect each other’s privacy and confidentiality by not mentioning each other’s presence in the group to other people. It is critically important that people respect each other’s decisions to share or keep this information private from others. So, please do not share information about anyone outside of this room.
4. Please respect each other’s opinions. There is no wrong or right way to feel or think here, and we want to encourage everyone to have the opportunity to share.
5. Please try to speak one-person at a time, so we can listen to what other colleagues have to say. This will also make it easier to transcribe our discussions accurately.
6. Finally, please maintain the COVID -19 protocols of social distancing, sanitizing and keeping your mask on throughout the discussion.

Are there any questions on the ground rules?

Thank you. I would now like everyone to introduce themselves. Please tell me:

1. Your name, or how you would like others to address you
2. Your age
3. How is your day going so far? What did you do today before this meeting?

Awareness and Demand

1. How did you first learn about the HIVST kits?
2. Can you still remember what you thought when you first learned about them?
   1. What were your emotions and thoughts?
   2. Did this information trigger you to think whether you could be at risk and whether this would be a good option to learn about your status?
   3. Did you believe that HIV self-testing would be possible?
3. Did someone come and speak to you about HIVST kits, or did you actively seek information yourself?
   1. [If from a third party]
      1. Who told you about HIVST kits?
      2. Did you trust this information? Please explain.
   2. [If they sought information themselves]
      1. Where did you search for information? Please explain.
4. Do you know if any of your peers, partner, friends, and family know about the HIVST kits?
   1. [If yes] What do they think about the kits?

Take Up

1. I have brought an HIVST kit with me today.Let’s talk about the packaging. What are your thoughts on the packaging?[Note: Please use a disinfectant towel to clean the surface before handing it to the participant - don’t take it back from the participant.]
   1. What do you think about the packaging?
   2. What do you think about the information provided in the packaging?
   3. Do you think anything is missing from the information provided in the packaging?
   4. Do you think anything should be removed from the information provided in the packaging?
   5. What would you change about the packaging?
2. Where are HIVST kits sold in your community?
3. Where would you feel comfortable buying this kit? Please explain.
4. [If they would not get the kit at a pharmacy] Would you feel comfortable buying an HIVST kit at your local pharmacy or drug shop? Please explain.
5. How much would you be willing to pay for an HIVST kit? Please explain.

Use

Now let’s open the package I gave you earlier. Take your time to look at it and to explore what is in the package.

1. What were you thinking when you first opened the test kit?
   1. Is there any particular reason you like or dislike the test kit?
   2. To what extent did you already know the information that was provided in the package? Please explain.
   3. Would you feel comfortable reading and interpreting your results, based on the instructions provided in this package?
   4. Are you confident in the next steps you must take after using the HIVST kit, per the instructions provided in this package?
      1. [If no] What other instruction would you need?
2. Suppose you purchased an HIVST kit. Where would you go or who would you ask if you had questions on using the kit?
3. Do you have any experience using other self-testing kits, such as malaria self-test kits [or rapid pregnancy test kit for women]?
   1. What has been your experience using these self-test kits?
      1. How often do you use these kits?
      2. How would you describe the ease of use?
      3. Do you trust the results provided by these kits? Please explain.
      4. Could you please describe the steps you follow after using the kits?
      5. Have you ever experienced a false positive? Please explain.
4. [If they used self-test kits in the past] How do you think the self-test kits you have used in the past would compare with HIVST kits?
   1. Would you trust the results more or less?
   2. Would you be willing to pay as much for an HIVST kit as you would for these other self-test kits?
   3. How would you compare the ease of use between the self-test kits you have used and the HIVST kit?
   4. Would you know what to do after taking the test?

Linkage to Care and Reporting

1. Would you be open to reporting your results to someone, such as a health care provider, in order to receive other health care services, or would you rather keep your results to yourself?
   1. [If they’re comfortable reporting their results] Who would you feel comfortable reporting your results to?
2. In general, what could be the positive effects of reporting back test results?
   1. And what could be negative consequences about reporting back test results?
