# Supplementary material for: Enablers and barriers to effective HIV self-testing in the private sector among sexually active youths in Nigeria: A qualitative study using journey map methodology
Source: PLoS One. 2023 Apr 27;18(4):e0285003. doi: 10.1371/journal.pone.0285003 (PMC10138200; doi:10.1371/journal.pone.0285003)
Supplement: Attachment — Submitted filename: PONE-Reviewers comment_BN1.pdf [file pone.0285003.s004.pdf]

1. The authors should streamline their manuscript to focus on Enablers and Barriers to effective HIV self-testing in Private sector.

2. Currently the manuscript is disorganized with irrelevant information,which doesn't address the enablers/barriers to effective HIVST.

3. Also the authors should explicitly describe how was the Journey Map method operationalized in this study so a reader can follow.

4. The author should also highlight what was the role of the private sector in this study. Did the author want to describe the role private health delivery systems?

5.The author should use scientific writing, currently the manuscript is full of irrelevant words which doesn't add any value to the paper. The authors should check for grammar and spellings before their next submission.
