# Supplementary material for: Enablers and barriers to effective HIV self-testing in the private sector among sexually active youths in Nigeria: A qualitative study using journey map methodology
Source: PLoS One. 2023 Apr 27;18(4):e0285003. doi: 10.1371/journal.pone.0285003 (PMC10138200; doi:10.1371/journal.pone.0285003)
Supplement: Attachment — Submitted filename: Response to Reviewers.docx [file pone.0285003.s003.docx]

Society for Family Health,

No. 8 Port Harcourt Crescent

Abuja, Nigeria

November 15^th^ 2022

The Editor-in-Chief,

Editorial Office

PLOS ONE

Dear Editor-in-Chief,

We thank the academic editor and the reviewers for the comments to improve on our manuscript [PONE-D-22-23769]. Please see below our itemized point-by-point responses has been highlighted. Moreover, in the revised manuscript, all changes have been tracked to indicate the revised sections. We would be happy to clarify any aspect of our responses if needed.

Best Regards,

Yusuf H. Wada

Corresponding Author

[hwada@sfhnigeria.org](mailto:hwada@sfhnigeria.org)

**Academic Editor**

1. Please ensure that your manuscript meets PLOS ONE's style requirements, including those for file naming. The PLOS ONE style templates can be found at

<https://journals.plos.org/plosone/s/file?id=wjVg/PLOSOne_formatting_sample_main_body.pdf> and <https://journals.plos.org/plosone/s/file?id=ba62/PLOSOne_formatting_sample_title_authors_affiliations.pdf>

We thank the academic editor for this comment, we have updated the manuscript with the PLOS ONE’s style requirement as advised.

2. Thank you for stating the following in the Acknowledgments Section of your manuscript:

“This study was possible by the generous support of Children’s Investment Fund Foundation (CIFF) - Global Fund partnership through the Population Services International (PSI). The contents herein are the sole responsibility of the authors and do not necessarily reflect the views of SFH, PSI or CIFF. The funders had no role in study design, data collection and analysis, decision to publish or preparation of the manuscript. We appreciate all members of the SFH SHIPS team, NACA, and ANAYD for their support during the data collection. We are most grateful to all the participants who took part in the study.”

We note that you have provided funding information that is not currently declared in your Funding Statement. However, funding information should not appear in the Acknowledgments section or other areas of your manuscript. We will only publish funding information present in the Funding Statement section of the online submission form.

Please remove any funding-related text from the manuscript and let us know how you would like to update your Funding Statement. Currently, your Funding Statement reads as follows:

“This study was possible by the generous support of Children’s Investment Fund Foundation (CIFF) - Global Fund partnership through the Population Services International (PSI). The contents herein are the sole responsibility of the authors and do not necessarily reflect the views of SFH, PSI or CIFF. The funders had no role in study design, data collection and analysis, decision to publish or preparation of the manuscript. “

Please include your amended statements within your cover letter; we will change the online submission form on your behalf

We thank the academic editor for this. This have been addressed.

3. Thank you for stating the following in your Competing Interests section:

“None”

Please complete your Competing Interests on the online submission form to state any Competing Interests. If you have no competing interests, please state "The authors have declared that no competing interests exist.", as detailed online in our guide for authors at <http://journals.plos.org/plosone/s/submit-now>

This information should be included in your cover letter; we will change the online submission form on your behalf.

We thank the academic editor for this. This have been addressed.

4. We note that you have stated that you will provide repository information for your data at acceptance. Should your manuscript be accepted for publication, we will hold it until you provide the relevant accession numbers or DOIs necessary to access your data. If you wish to make changes to your Data Availability statement, please describe these changes in your cover letter and we will update your Data Availability statement to reflect the information you provide.

We thank the academic editor for this.

5. Please ensure that you refer to Figure 1 in your text as, if accepted, production will need this reference to link the reader to the figure.

We thank the academic editor for this. We have changed in figure to a new one as reflected in the fig 1 uploaded.

6. We note that Figure 1 in your submission contain [map/satellite] images which may be copyrighted. All PLOS content is published under the Creative Commons Attribution License (CC BY 4.0), which means that the manuscript, images, and Supporting Information files will be freely available online, and any third party is permitted to access, download, copy, distribute, and use these materials in any way, even commercially, with proper attribution. For these reasons, we cannot publish previously copyrighted maps or satellite images created using proprietary data, such as Google software (Google Maps, Street View, and Earth). For more information, see our copyright guidelines: <http://journals.plos.org/plosone/s/licenses-and-copyright>.

We require you to either (1) present written permission from the copyright holder to publish these figures specifically under the CC BY 4.0 license, or (2) remove the figures from your submission:

a. You may seek permission from the original copyright holder of Figure(s) [#] to publish the content specifically under the CC BY 4.0 license.

We recommend that you contact the original copyright holder with the Content Permission Form (<http://journals.plos.org/plosone/s/file?id=7c09/content-permission-form.pdf>) and the following text:

“I request permission for the open-access journal PLOS ONE to publish XXX under the Creative Commons Attribution License (CCAL) CC BY 4.0 (<http://creativecommons.org/licenses/by/4.0/>). Please be aware that this license allows unrestricted use and distribution, even commercially, by third parties. Please reply and provide explicit written permission to publish XXX under a CC BY license and complete the attached form.”

Please upload the completed Content Permission Form or other proof of granted permissions as an "Other" file with your submission.

In the figure caption of the copyrighted figure, please include the following text: “Reprinted from [ref] under a CC BY license, with permission from [name of publisher], original copyright [original copyright year].”

b. If you are unable to obtain permission from the original copyright holder to publish these figures under the CC BY 4.0 license or if the copyright holder’s requirements are incompatible with the CC BY 4.0 license, please either i) remove the figure or ii) supply a replacement figure that complies with the CC BY 4.0 license. Please check copyright information on all replacement figures and update the figure caption with source information. If applicable, please specify in the figure caption text when a figure is similar but not identical to the original image and is therefore for illustrative purposes only.

The following resources for replacing copyrighted map figures may be helpful:

USGS National Map Viewer (public domain): [http://viewer.nationalmap.gov/viewer/](https://eur03.safelinks.protection.outlook.com/?url=http%3A%2F%2Fviewer.nationalmap.gov%2Fviewer%2F&data=05%7C01%7Chwada%40sfhnigeria.org%7Ca1b23eadac054473f0d408dabdde84c7%7C064cb1ce397a4f87b199216ae587dccc%7C0%7C0%7C638031060669790322%7CUnknown%7CTWFpbGZsb3d8eyJWIjoiMC4wLjAwMDAiLCJQIjoiV2luMzIiLCJBTiI6Ik1haWwiLCJXVCI6Mn0%3D%7C3000%7C%7C%7C&sdata=2kRogFXwTudC0vh46stQOv5k%2Fg2QxFCPaXvdVX%2FbSQ8%3D&reserved=0)

The Gateway to Astronaut Photography of Earth (public domain): [http://eol.jsc.nasa.gov/sseop/clickmap/](https://eur03.safelinks.protection.outlook.com/?url=http%3A%2F%2Feol.jsc.nasa.gov%2Fsseop%2Fclickmap%2F&data=05%7C01%7Chwada%40sfhnigeria.org%7Ca1b23eadac054473f0d408dabdde84c7%7C064cb1ce397a4f87b199216ae587dccc%7C0%7C0%7C638031060669790322%7CUnknown%7CTWFpbGZsb3d8eyJWIjoiMC4wLjAwMDAiLCJQIjoiV2luMzIiLCJBTiI6Ik1haWwiLCJXVCI6Mn0%3D%7C3000%7C%7C%7C&sdata=gyoCvas0zRmOjUbioqh7yjpHw5o2xnVge%2Fr9keRne8U%3D&reserved=0)

Maps at the CIA (public domain): <https://www.cia.gov/library/publications/the-world-factbook/index.html> and [https://www.cia.gov/library/publications/cia-maps-publications/index.html](https://eur03.safelinks.protection.outlook.com/?url=https%3A%2F%2Fwww.cia.gov%2Flibrary%2Fpublications%2Fcia-maps-publications%2Findex.html&data=05%7C01%7Chwada%40sfhnigeria.org%7Ca1b23eadac054473f0d408dabdde84c7%7C064cb1ce397a4f87b199216ae587dccc%7C0%7C0%7C638031060669790322%7CUnknown%7CTWFpbGZsb3d8eyJWIjoiMC4wLjAwMDAiLCJQIjoiV2luMzIiLCJBTiI6Ik1haWwiLCJXVCI6Mn0%3D%7C3000%7C%7C%7C&sdata=qgKnB9FA0qRfdUB0AzWf55Iyskug6O%2FcH3XKKW5yo38%3D&reserved=0)

NASA Earth Observatory (public domain): <http://earthobservatory.nasa.gov/>

Landsat: [http://landsat.visibleearth.nasa.gov/](https://eur03.safelinks.protection.outlook.com/?url=http%3A%2F%2Flandsat.visibleearth.nasa.gov%2F&data=05%7C01%7Chwada%40sfhnigeria.org%7Ca1b23eadac054473f0d408dabdde84c7%7C064cb1ce397a4f87b199216ae587dccc%7C0%7C0%7C638031060669790322%7CUnknown%7CTWFpbGZsb3d8eyJWIjoiMC4wLjAwMDAiLCJQIjoiV2luMzIiLCJBTiI6Ik1haWwiLCJXVCI6Mn0%3D%7C3000%7C%7C%7C&sdata=Lux7W3EykucGSONhZSPL85faoVmkpJ3yG8fsPTatcyA%3D&reserved=0)

USGS EROS (Earth Resources Observatory and Science (EROS) Center) (public domain): [http://eros.usgs.gov/#](https://eur03.safelinks.protection.outlook.com/?url=http%3A%2F%2Feros.usgs.gov%2F%23&data=05%7C01%7Chwada%40sfhnigeria.org%7Ca1b23eadac054473f0d408dabdde84c7%7C064cb1ce397a4f87b199216ae587dccc%7C0%7C0%7C638031060669790322%7CUnknown%7CTWFpbGZsb3d8eyJWIjoiMC4wLjAwMDAiLCJQIjoiV2luMzIiLCJBTiI6Ik1haWwiLCJXVCI6Mn0%3D%7C3000%7C%7C%7C&sdata=cpWxpfNrYGbg4nD3lODFvwgijDzLmZErbxprPKCAI5U%3D&reserved=0)

Natural Earth (public domain): [http://www.naturalearthdata.com/](https://eur03.safelinks.protection.outlook.com/?url=http%3A%2F%2Fwww.naturalearthdata.com%2F&data=05%7C01%7Chwada%40sfhnigeria.org%7Ca1b23eadac054473f0d408dabdde84c7%7C064cb1ce397a4f87b199216ae587dccc%7C0%7C0%7C638031060669790322%7CUnknown%7CTWFpbGZsb3d8eyJWIjoiMC4wLjAwMDAiLCJQIjoiV2luMzIiLCJBTiI6Ik1haWwiLCJXVCI6Mn0%3D%7C3000%7C%7C%7C&sdata=eQfoeruWrq1Tn8H4HFxyyJgW11Yowp1MZkGkULiBrGo%3D&reserved=0)
[Note: HTML markup is below. Please do not edit.]

We thank the academic editor for this. We have provided a replacement map as directed.

Reviewers' comments:
Reviewer's Responses to Questions

**Comments to the Author**
**1. Is the manuscript technically sound, and do the data support the conclusions?**
**The manuscript must describe a technically sound piece of scientific research with data that supports the conclusions. Experiments must have been conducted rigorously, with appropriate controls, replication, and sample sizes. The conclusions must be drawn appropriately based on the data presented.**

Reviewer #1: Yes

Reviewer #2: Partly

2. Has the statistical analysis been performed appropriately and rigorously?

Reviewer #1: N/A

Reviewer #2: Yes

3. Have the authors made all data underlying the findings in their manuscript fully available?
The [PLOS Data policy](https://eur03.safelinks.protection.outlook.com/?url=http%3A%2F%2Fwww.plosone.org%2Fstatic%2Fpolicies.action%23sharing&data=05%7C01%7Chwada%40sfhnigeria.org%7Ca1b23eadac054473f0d408dabdde84c7%7C064cb1ce397a4f87b199216ae587dccc%7C0%7C0%7C638031060669790322%7CUnknown%7CTWFpbGZsb3d8eyJWIjoiMC4wLjAwMDAiLCJQIjoiV2luMzIiLCJBTiI6Ik1haWwiLCJXVCI6Mn0%3D%7C3000%7C%7C%7C&sdata=6IoJaX4HUQ8lvRRRKSqKWGOS25kSsBuYEKoPFbsPtjY%3D&reserved=0) requires authors to make all data underlying the findings described in their manuscript fully available without restriction, with rare exception (please refer to the Data Availability Statement in the manuscript PDF file). The data should be provided as part of the manuscript or its supporting information, or deposited to a public repository. For example, in addition to summary statistics, the data points behind means, medians and variance measures should be available. If there are restrictions on publicly sharing data—e.g. participant privacy or use of data from a third party—those must be specified.

Reviewer #1: No

Reviewer #2: Yes

4. Is the manuscript presented in an intelligible fashion and written in standard English?
PLOS ONE does not copyedit accepted manuscripts, so the language in submitted articles must be clear, correct, and unambiguous. Any typographical or grammatical errors should be corrected at revision, so please note any specific errors here.

Reviewer #1: No

Reviewer #2: No

5. Review Comments to the Author
Please use the space provided to explain your answers to the questions above. You may also include additional comments for the author, including concerns about dual publication, research ethics, or publication ethics. (Please upload your review as an attachment if it exceeds 20,000 characters)

Reviewer #1:

“Enablers and Barriers to Effective HIV Self-testing in the Private Sector among Sexually active youths in Nigeria: A Qualitative Study using Journey Map methodology” presents qualitative data from in-depth interviews and focus group discussions with 80 participants involved in market research in Lagos, Anambra, and Kano states from January 2021 to October 2021. Although this is an important area of investigation, the current writing is weak and has numerous grammatical errors, making portions of the manuscript difficult to comprehend. I strongly recommend utilizing the services of a copyeditor to enhance clarity. Some of my questions, concerns, and suggestions are as follows:

We thank the reviewer 1 for this comment. The manuscript has been edited by a copyeditor to enhance clarity as advised.

1. Abstract – The opening sentences in the objectives and methods are very similar. Please revise the objectives to specify that the purpose of this qualitative study was to identify enablers and barriers to the uptake of HIV self-testing among sexually active youth in Nigeria using journey map methodology.

We thank the reviewer 1 for this comment, the objectives of the study have been rephrased as advised.

2. Introduction – It would be helpful to clarify the meaning of “effective self-testing in the private sector” in the introduction. Does this refer to the distribution of HIV self-testing kits by manufacturers or pharmacies? Would the kits be available for free, or would they have to be purchased? Is this already being done in any states in Nigeria? What types of HIV self-tests are commercially available – oral fluid or finger-stick blood or both?
We thank reviewer 1 for this comment. Within the context of this manuscript, effective self-testing in the private sector means the effective utilization of HIVST kits and linkage to sexual reproductive health services and to HIV prevention by pharmacies (who are only approved to provide those services), while the distribution of HIV self-testing at the retail level is by the pharmacies. This is in line with the revised National HIV and AIDS strategic framework 2019-2021 as a priority policy and porgarmmatic approach to HIV response in Nigeria (NACA. Revised National HIV and AIDS Strategic Framework 2019-2021. Abuja: Nigeria. National Agency for Control of AIDS; 2019). We also updated the manuscript to reflect the context of effective self-testing in the private sector. The kits would have to be purchased at the pharmacies and currently being sold in different pharmacies in Lagos, FCT, Rivers, Akwa-Ibom, Anambra and other states in Nigeria. Therefore, this pilot study finding is set to to provide evidencebased date and influence government, policy makers, donors and business/investment case to scale up free, incentivized or subsidized HIVST kits and for successful future HIVST campaigns in the private sector. There are many types of HIVST kits commercially available in Nigeria such as OraQuick (oral-fluid based), Mylan (blood-based), Insti (blood-based), DrGregs (blood based) and 3-H viral blood check (blood-based). There have been some discrepancies from regulatory bodies on which is approved or not, reason we didn’t discuss earlier in our introduction. All comment raised by the reviewer have been inputted in the introduction part of the manuscript (line 77-84).
3. Methods – How were participants recruited? What were the inclusion and exclusion criteria?
We thank reviewer 1 for this comment. The recruitment of participant was using a clearly spelt-out eligibility criteria (see below) and using a convenience sampling from a list of participants who fulfilled the criteria using a CBOs & CSOs drafted list and were willing to write an informed consent, which was carried out in accordance with ethical guidelines of 1975 declaration of Hesinki Declaration of 1975, revised 2000.

Inclusion criteria were being either male or female, aged between 18 and 29, being sexually active, currently a resident of urban or peri urban setting in Nigeria, has ever taken an HIVST and other group who has never taken an HIVST, willing and able to provide verbal oral informed consent, and willing to consent to an audio recorded session.

We have updated the participant selection and recruitment part to reflect the comment being raised (line 125-133).
4. Methods – Please provide a brief description (2-3 sentences) of the journey map methodology along with appropriate citations for the benefit of readers unfamiliar with this approach.

We thank reviewer 1 for this comment. We have updated the method with a journey map section as recommended.
5. Results – It would be helpful to include a table summarizing the demographic characteristics of the 80 participants. Figure 1 does include their geographic distribution, but no figures have been presented. For example, how many men and women were in the sample? What was the age range? How many lived in urban and peri-urban areas?
We thank reviewer 1 for this comment. We have updated the result section with (Table 1) to reflect the gender, age range and geographic distribution.
6 Discussion – Is there a reason why the results might not be subject to social desirability bias?

We thank the reviewer for this comment. We have already reported in our limitations that our study might be subject to social desirability bias, but we try as much to reduce it by using indirect questions, self-completion, the use of proxy users (both the users and non-users), and random sampling from list of participants presented. This have also been updated in the manuscript.

Reviewer #2: The authors should streamline their manuscript to focus on Enablers and Barriers to effective HIV self-testing in Private sector. Currently the manuscript is disorganized with irrelevant information, which doesn't address the enablers/barriers to effective HIVST.

We thank the reviewer 2 for the comment. We have ie

Also, the authors should explicitly describe how was the Journey Map method operationalized in this study so a reader can follow.

We thank reviewer 2 for this comment. This have been reflected in the methodology section by adding a journey map method, how participants were recruited and the inclusion and exclusion criteria.

The author should also highlight what was the role of the private sector in this study. Did the author want to describe the role private health delivery systems?

We thank the reviewer 2 for this comment. We have updated the introduction section to reflect the context of private sector in this study.

The author should use scientific writing, currently the manuscript is full of irrelevant words which doesn't add any value to the paper. The authors should check for grammar and spellings before their next submission.

We thank the reviewer for this comment. We have employed the service of a copy writer and now reflect in the updated manuscript.

6. PLOS authors have the option to publish the peer review history of their article ([what does this mean?](https://eur03.safelinks.protection.outlook.com/?url=https%3A%2F%2Fjournals.plos.org%2Fplosone%2Fs%2Feditorial-and-peer-review-process%23loc-peer-review-history&data=05%7C01%7Chwada%40sfhnigeria.org%7Ca1b23eadac054473f0d408dabdde84c7%7C064cb1ce397a4f87b199216ae587dccc%7C0%7C0%7C638031060669946571%7CUnknown%7CTWFpbGZsb3d8eyJWIjoiMC4wLjAwMDAiLCJQIjoiV2luMzIiLCJBTiI6Ik1haWwiLCJXVCI6Mn0%3D%7C3000%7C%7C%7C&sdata=EmCTUAN9K87ym3zq1AsglodtilKfDQSsssYWq0N6fSQ%3D&reserved=0)). If published, this will include your full peer review and any attached files. If you choose “no”, your identity will remain anonymous but your review may still be made public.

Do you want your identity to be public for this peer review? For information about this choice, including consent withdrawal, please see our [Privacy Policy](https://eur03.safelinks.protection.outlook.com/?url=https%3A%2F%2Fwww.plos.org%2Fprivacy-policy&data=05%7C01%7Chwada%40sfhnigeria.org%7Ca1b23eadac054473f0d408dabdde84c7%7C064cb1ce397a4f87b199216ae587dccc%7C0%7C0%7C638031060669946571%7CUnknown%7CTWFpbGZsb3d8eyJWIjoiMC4wLjAwMDAiLCJQIjoiV2luMzIiLCJBTiI6Ik1haWwiLCJXVCI6Mn0%3D%7C3000%7C%7C%7C&sdata=C5ElmeWihT2rtOgIuJ%2BfBPYHsoErm1tDBa%2B2xKDaiS8%3D&reserved=0).

Reviewer #1: No

Reviewer #2: No
