# Supplementary material for: Enablers and barriers to effective HIV self-testing in the private sector among sexually active youths in Nigeria: A qualitative study using journey map methodology
Source: PLoS One. 2023 Apr 27;18(4):e0285003. doi: 10.1371/journal.pone.0285003 (PMC10138200; doi:10.1371/journal.pone.0285003)
Supplement: Attachment — Submitted filename: Response to reviewers.docx [file pone.0285003.s005.docx]

Society for Family Health,

No. 8 Port Harcourt Crescent

Abuja, Nigeria

March 30^th^ 2023

The Editor-in-Chief,

Editorial Office

PLOS ONE

Dear Editor-in-Chief,

We thank the academic editor and the reviewers for the comments to improve on our manuscript [PONE-D-22-23769]. Please see below our itemized point-by-point responses has been highlighted. Moreover, in the revised manuscript, all changes have been tracked to indicate the revised sections. We would be happy to clarify any aspect of our responses if needed.

Best Regards,

Yusuf H. Wada

Corresponding Author

[hwada@sfhnigeria.org](mailto:hwada@sfhnigeria.org)

**Reviewer #2:**

The authors should streamline their manuscript to focus on Enablers and Barriers to effective HIV self-testing in Private sector. Currently the manuscript is disorganized with irrelevant information, which doesn't address the enablers/barriers to effective HIVST. Also, the authors should explicitly describe how was the Journey Map method operationalized in this study so a reader can follow. The author should also highlight what was the role of the private sector in this study. Did the author want to describe the role private health delivery systems? The author should use scientific writing, currently the manuscript is full of irrelevant words which doesn't add any value to the paper. The authors should check for grammar and spellings before their next submission.

We thank the reviewer for this comment. We have reviewed how the Journey map was operationalized (line 112-125), describe the role of private sector in the manuscript, reviewed by a native speaker for irrelevant word and updated the format/logical flow using the COREQ checklist which was adopted from Tong A, Sainsbury P, Craig J. Consolidated criteria for reporting qualitative research (COREQ): a 32-item checklist for interviews and focus groups. International Journal for Quality in Health Care. 2007. Volume 19, Number 6: pp. 349 – 357 as highlighted by the other reviewer.

Reviewer #3:

In their present manuscript, the authors have documented factors likely to influence young people's uptake of HIV self-testing in Nigeria through a qualitative approach. The objective is clear and a timely question in the context of HIV prevention in West Africa. The method is appropriate, and the authors have correctly addressed previous reviewer suggestions. Here are a few comments to consider to further strengthen the manuscript:
•The authors should ensure that the paper is formatted according to the COREQ checklist.
We thank the reviewer for this comment. We have formatted the manuscript according to the COREQ checklist and updated as appropriate. The COREQ Checklist was adopted from Tong A, Sainsbury P, Craig J. Consolidated criteria for reporting qualitative research (COREQ): a 32-item checklist for interviews and focus groups. International Journal for Quality in Health Care. 2007. Volume 19, Number 6: pp. 349 – 357.
Methods:
•Consider moving the “Recruitment and participants selection” section after the “Journey map operation” section.

We thank the reviewer for this comment. We have revised as advised with the recruitment and participants selection after the journey map operation section.
•Line 121: Describe the HIV prevention stage. Refer to the following articles that describes HIV prevention continuum. A. McNairy, Margaret L, and Wafaa M El-Sadr. “A paradigm shift: focus on the HIV prevention continuum.” Clinical infectious diseases: an official publication of the Infectious Diseases Society of America vol. 59 Suppl 1,Suppl 1 (2014): S12-5. doi:10.1093/cid/ciu251. B. Horn, Tim et al. “Towards an integrated primary and secondary HIV prevention continuum for the United States: a cyclical process model.” Journal of the International AIDS Society vol. 19,1 21263. 17 Nov. 2016, doi:10.7448/IAS.19.1.21263.

We thank the reviewer for this comment. We have referred to the article and revised as appropriate (see line 126-131 and reference 18-20).
•Where was the interview conducted and by whom?

We thank the reviewer for this comment. We already have that captured in the manuscript (line 157- 162). We have also included a sentence and updated the manuscript on who conducted the interview and by whom.

“The project team from SFH and Busara who were part of the face validity team carried out the qualitative research and collected data in the four dominant language of Nigeria (English, Igbo, Hausa, and Yoruba) using the IDIs and FGDs guides. IDIs were done in English over the phone, while FGDs were conducted in-person for the sexually active youths using the four languages in the three states. We conducted gender-specific FGDs - one FGD was either all men or all women, but we mixed HIVST users and HIVST non-users. During both the IDIs and FGDs, we asked respondents open-ended questions regarding their opinions and perceptions of HIVST, and for IDIs with HIVST users, we asked questions about their past experiences with taking up and using HIVST kits”.

•Include more description about the study setting. Anambra, Kano and Lagos states. Perhaps mention that these states were identified as PEPFAR priority states given evidence of high HIV burden and unmet needs for HIV/AIDS treatment services

We thank the reviewer for this comment. We have included the brief description of the study setting and identify them as PEPFAR priority states and other supporting information as advised (see line 110-117).
•How was data triangulated?
We thank the reviewer for this comment. We have included how the data was triangulated in our manuscript as advised (see line 167-170).

Result
•Consider deleting “The journey map is the process with each stage having its own characteristics using the person’s experience to identify problems and suggest an improvement. However, the person perceives this as a real journey that can be used to improve each stage of HIV self-testing.” This has already been described in the methods section.

We thank the reviewer for this comment. This have been revised and deleted this section.
•Line 213 revise to “While the barriers identified at the attracting stage.”

We thank the reviewer for this comment. We have revised as advised.
•Line 250, include the dollar equivalence of the naira

We thank the reviewer for this comment. We have included the dollar equivalence of the naira (US$ 0.2-2).
